# Efficacy and safety of oral minoxidil in the treatment of alopecia: a single-arm rate meta-analysis and systematic review

**DOI:** 10.3389/fphar.2025.1556705

**Published:** 2025-06-03

**Authors:** Chang Liu, Xiaojie Liu, Ting Shi, Yun Wang, Chaoyang Sui, Wenan Zhang, Bowen Wang

**Affiliations:** Department of Burns and Plastic Surgery, Yantaishan Hospital, Yantai, China

**Keywords:** minoxidil, alopecia, efficacy, safety, meta-analysis

## Abstract

**Background:**

Hair loss is a prevalent aesthetic concern that impacts the quality of life and self-image of numerous individuals. While topical minoxidil has been extensively utilized in addressing hair loss for several decades, the effectiveness and safety of oral minoxidil are still a topic of debate. Despite its use in certain clinical settings, the precise efficacy and safety of oral minoxidil have not been conclusively validated. Hence, conducting a one-arm rate meta-analysis and systematic review to assess the efficacy and safety of oral minoxidil in treating alopecia holds significant importance. This endeavor aims to furnish additional evidence to support clinical practice and offer guidance for future research in this domain.

**Methods:**

We conducted a comprehensive search on electronic databases including PubMed, Embase, Cochrane Library, and Web of Science from their inception up to 1 October 2024. The primary objective was to assess both the efficacy (measured by the degree of symptom improvement) and safety (including adverse event incidence) of oral minoxidil in the treatment of hair loss.

**Results:**

A total of 2,933 patients from 27 studies were included in the analysis. The efficacy of oral minoxidil for treating alopecia was primarily assessed based on the degree of symptom improvement. Among the participants, 35% (95% CI: 22%–49%) experienced significant symptom improvement, 47% (95% CI: 38%–55%) showed symptom improvement, and 26% (95% CI: 16%–37%) had stable symptoms. The incidence of adverse events in the safety evaluation was reported at 27% (95% CI: 18%–36%).

**Conclusion:**

This systematic review and meta-analysis indicate that individuals with hair loss may derive benefits from oral minoxidil, particularly at doses exceeding 1 mg. Nonetheless, additional research or data is essential to definitively establish its efficacy and safety.

**Systematic Review Registration:**

https://www.crd.york.ac.uk/PROSPERO/, identifier CRD42024581183.

## Introduction

Hair loss is a prevalent cosmetic issue that impacts over 50% of adults at some point in their lives (Gan and Sinclair, 2005). Between 1990 and 2019, the worldwide occurrence of alopecia areata and the related disease-adjusted life years (DALY) both rose by around 49% (Wang et al., 2022). This trend may be correlated with shifts in modern lifestyles, heightened stress levels, and the impact of environmental factors. The occurrence of hair loss is intricately linked to age and gender. As individuals age, both men and women are more susceptible to experiencing hair loss, with androgenic alopecia being notably more prevalent among older men. Hair loss is not merely a physical concern; it can also significantly impact an individual’s mental wellbeing. The lack of acceptance regarding hair loss often leads to reduced self-esteem and social anxiety (Hair, 2005; Dika et al., 2016). Consequently, the development of effective treatment strategies is of paramount importance.

Hair loss encompasses various types, with the most prevalent being: male pattern hair loss, female pattern alopecia, alopecia areata, and telogen effluvium. At present, the standard first-line treatment for androgenic alopecia (AGA) includes topical minoxidil (2%–5%), oral finasteride, and low-level laser therapy. Oral formulations of minoxidil were initially utilized in general medicine to address severe and uncontrolled high blood pressure at doses of 10–40 mg (Gilmore et al., 1970). Interestingly, early trials of oral minoxidil as an antihypertensive drug inadvertently documented side effects of long-term use, such as hypertrichosis and hirsutism, and also reported the drug’s potential to stimulate hair growth (Hagstam et al., 1982; Devine et al., 1977; Burzi et al., 2021). In recent years, there has been a surge in clinical studies examining the impact of oral minoxidil on male pattern baldness. Among these studies, a pivotal one sought to compare the effects of oral minoxidil with topical minoxidil, aiming to evaluate their efficacy and safety in treating male pattern hair loss. In a randomized controlled trial, researchers juxtaposed 1 mg of oral minoxidil with 5% topical minoxidil for the treatment of male pattern baldness. The findings revealed that both treatments exhibited similar effects in fostering hair growth. Noteworthy conclusions from this study encompass (Penha et al., 2024): (1) Hair growth effects: Patients treated with oral minoxidil and topical minoxidil experienced significant improvements in hair density and growth rate; (2) Safety: Individuals administered with 1 mg of oral minoxidil did not encounter serious adverse reactions throughout the treatment period, indicating a high level of safety. The aim of this meta-analysis was to offer substantiated evidence regarding the effectiveness and safety of oral minoxidil in addressing hair loss, drawing from available data.

## Methods

This systematic evaluation and meta-analysis follows the Preferred Reporting Items for Systematic Evaluation and Meta-Analysis (PRISMA) (Page et al., 2021) statement and has been pre-registered with PROSPERO under registration number CRD42024581183.

### Search Strategy and study selection

We systematically searched PubMed, Embase, Cochrane Library, and Web of Science databases to identify studies published before 1 October 2024, related to the use of oral minoxidil in treating patients with alopecia areata. Search terms included “oral minoxidil,” “alopecia,” and similar subject terms. Additionally, we reviewed references of these articles to identify supplementary resources.

### Selection criteria and data extraction

Two authors independently assessed all records against inclusion and exclusion criteria. They managed and organized all relevant records using EndNote X9 software. After removing duplicate records, potentially eligible clinical trials underwent independent full-text review. Inclusion of the full text of potential clinical studies was based on the following PICOS criteria: (1) Participants were alopecia patients aged 18 years or older with a medically confirmed diagnosis; (2) Studies were cohort, randomized controlled, case-control, or propensity score matching studies; (3) The intervention group received oral minoxidil treatment; (4) Studies provided adequate data for calculating efficacy and/or safety outcomes. Exclusion criteria comprised: (1) Studies with fewer than 10 participants; (2) Inadequate statistical data; (3) Duplicate publications.

The data extraction process adhered to the PRISMA 2020 guidelines, which provided comprehensive guidance throughout. Abstracts and full-text articles underwent rigorous screening by two independent authors using predefined inclusion and exclusion criteria. For each eligible study, a range of key details were documented, encompassing the first author, year of publication, article type, study type, treatment type, hair loss pattern among patients, total patient count, average duration of drug use, degree of symptom improvement, incidence of adverse effects, and other relevant parameters.

### Statistical analysis

We primarily utilized Review Manager version 5.4 (RevMan), a professional software provided by the Cochrane Collaboration, to conduct our analyses. Given that most of the included studies were single-arm clinical trials with diverse rates as the primary outcome measures, our research team employed non-comparative binary data in RevMan software for meta-analysis. Dominant odds ratios (OR) and their respective 95% confidence intervals (CI) served as the evaluation indices. Heterogeneity was assessed using the χ2 test and I^2^ test. Sensitivity analysis involved sequentially removing included studies to ensure that each individual trial did not significantly impact the overall outcome. In cases of significant heterogeneity, we employed the random effects model, with the option to use a fixed-effect model as an alternative. Statistical significance was set at P < 0.05. A Higgins I^2^ statistic <50% indicated low heterogeneity, while a Higgins I^2^ statistic >50% indicated high heterogeneity. Subgroup analyses were conducted to identify sources of heterogeneity and factors associated with clinical outcomes.

### Study quality

Two reviewers used the MINORS scale to assess study quality. The scale was designed to assess non-randomized studies and consisted of eight items, each rated on a scale ranging from 0–2, for a total score of 16. High-quality studies were defined as those with a score of 13–16 (rigorous study design, complete information, low risk of bias, high reliability and validity of results, and able to provide strong evidence for clinical practice or decision-making), while studies with a score of 9–12 were considered to be of moderate quality (study design was more reasonable, necessary information was provided, but some limitations or potential sources of bias still existed, reliability of results and validity are somewhat assured but need to be interpreted with caution). Studies with a score of less than 9 were considered to be of low quality (significant flaws in study design, key information missing, high risk of bias, reliability and validity of results seriously compromised) and were therefore excluded.

## Results

### Characteristics of included studies

**FIGURE 1:**
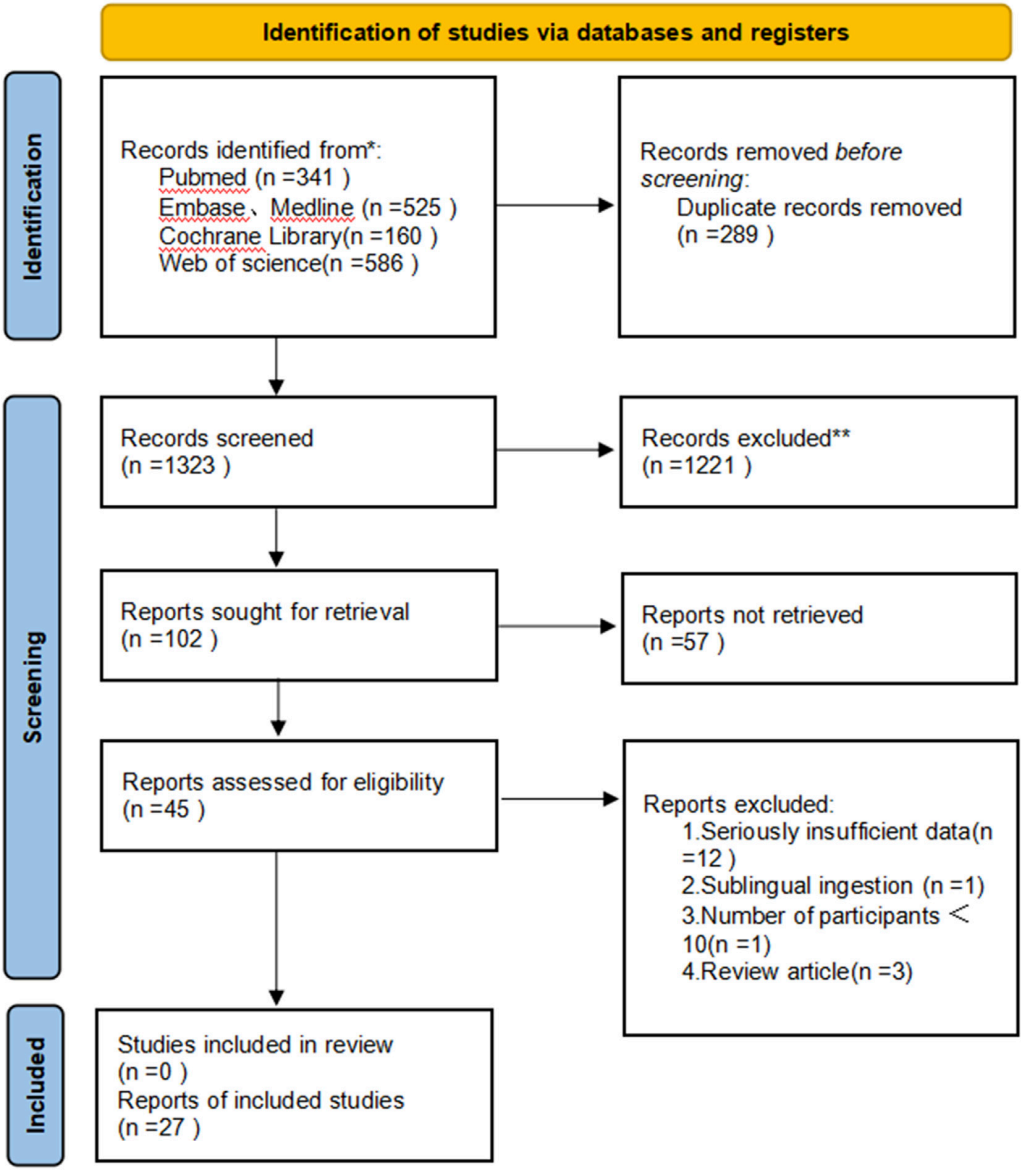
Preferred Reporting Items for Systematic Reviews and Meta-Analyses (PRISMA) diagram of the study selection.

We performed an extensive search and retrieved 1612 articles. Following the removal of 289 duplicate articles, we were left with 1,323 unique articles. Subsequently, after reviewing the titles and abstracts, we identified 45 articles for in-depth analysis. Upon conducting full-text screening, 27 papers met the inclusion criteria and were incorporated into our systematic review. The meta-analysis comprised 17 single-arm clinical studies (Beach, 2018; Jimenez-Cauhe et al., 2019; Pirmez and Salas-Callo, 2020; Wambier et al., 2021; Jha et al., 2020; Panchaprateep and Lueangarun, 2020; Rodrigues-Barata et al., 2020; Vastarella et al., 2020; Beach et al., 2020; Sanabria et al., 2021; Vañó-Galván et al., 2021a; Vañó-Galván et al., 2021b; Yin et al., 2022; Imhof et al., 2023; Iorizzo et al., 2023; Bloch and Carlos, 2024) and 10 randomized controlled trials (Penha et al., 2024; Ramos et al., 2019; Jha et al., 2021; Vahabi-Amlashi et al., 2021; Nascimento e Silva et al., 2022; Villarreal-Villarreal et al., 2022; Feaster et al., 2023; Minta et al., 2023; Asilian et al., 2024; Janaani et al., 2024), encompassing a total of 2933 patients. The primary characteristics of the included studies are outlined in Tables 1, 2. Additionally, we documented the total number of studies screened, selected, and excluded in a prismatic flow chart (Figure 1).

**TABLE 1:** Study characteristics of oral minoxidil in the treatment of alopecia.

Author year	Article type	Research type	Methods of treatment	Hair loss type	No. of patients
Renée A Beach2018	Letter	single-arm	Minoxidil 1.25 mg	AGA/TA/FFA/Seb D/TE	20
Juan Jimenez-Cauhe2019	Letter	single-arm	Minoxidil 2.5/5 mg	AGA	41
Paulo Müller Ramos2019	Letter	RCT	Minoxidil 1 mg	FPHL	26
R. Pirmez2019	Letter	single-arm	Minoxidil 0.25 mg	AGA	25
Carlos G. Wambier2019	Letter	single-arm	Tofacitinib + Minoxidil 5–10 mg	AA	12
Abhijeet Jha2020	Letter	single-arm	Minoxidil 1.25 mg	AGA	32
Ratchathorn Panchaprateep2020	Article	single-arm	Minoxidil 5 mg	AGA	30
Rita Rodrigues-Barata2020	Letter	single-arm	Minoxidil 0.25/0.5/1/2 mg + Oral dutasteride/Mesotherapy with Dutasteride/Topical 5% minoxidil/Platelet-rich plasma/Oral finasteride/Oral flutamide/Oral bicalutamide/Oral cyproterone acetate/Low-level light therapy/Topical latanoprost	FPHL	148
Maria Vastarella2020	Article	single-arm	Minoxidil 1.5–2 mg	FAA	12
Renée A. Beach 2020	Letter	single-arm	Minoxidil 2.5 mg	AGA/FFA/CCCA/LPP/TE	51
Baltazar Sanabria2020	Letter	single-arm	Minoxidil <5 mg	AGA	435
Sergio Vano-Galvan2020	Letter	single-arm	Minoxidil 0.25–1 mg	LPP	51
Abhijeet Kumar Jha2021	Article	RCT	Platelet-rich plasma + Minoxidil 2.5 mg	AGA	47
Sadegh Vahabi-Amlashi2021	Article	RCT	Minoxidil 0.25 mg	FPHL	36
Sergio Van ∼ o-Galvan2021	Article	single-arm	Minoxidil Average 1.63 mg	AA/LPP/FFA/TE	1404
Marcella Nascimento E Silva2022	Letter	RCT	Minoxidil 0.25/1 mg	FPHL	14
C.D. Villarreal-Villarreal2022	letter	RCT	Local microinjection of dutasteride + Minoxidil Average 4.36 mg	AGA	47
L. Yin2022	letter	single-arm	Minoxidil 0.625/1.25/2.5/5 mg	AGA	60
Reese Imhof2023	Meeting Abstract	single-arm	Minoxidil 2.5 mg	AA	40
Brittany Feaster 2023	Article	RCT	Minoxidil 0.625–2.5 mg	AA/TE	105
Matilde Iorizzo2023	Letter	single-arm	Minoxidil 1.5 mg	PCIA	15
Abena Minta2023	Letter	RCT	Minoxidil 1.25 mg	PCIA	13
Ali Asilian2023	Article	RCT	Minoxidil 1 mg	AA	33
Leila David Bloch2024	Article	single-arm	Minoxidil 0.5/1/2/2.5/3.5/5 mg	AGA	100
Deesha D. Desai2024	Letter	single-arm	Minoxidil 1.49 mg	AGA	69
Janaani, P2024	Meeting Abstract	RCT	Minoxidil 2.5 mg	AGA	22
Mariana Alvares Penha2024	Article	RCT	Minoxidil 5 mg	AGA	45

AGA, androgenetic alopecia; FFA, frontal fibrosing alopecia; Seb D = seborrheic dermatitis; TA, traction alopecia; TE, telogen effluvium; FPHL, Female pattern hair loss; AA, alopecia areata; FAA, Female Androgenetic Alopecia; LPP, Lichen planopilaris; CCCA, Central centrifugal cicatricial alopecia; PCIA, Persistent chemotherapy-induced alopecia.

**TABLE 2:** Main features included in the studies.

Author year	Average Time spent	Symptoms significantly improved	Symptom improvement	Symptom stabilization	Adverse event rate	Rate of overhair	Incidence of lower extremity edema
Renée A Beach2018	6 Months	—	5/18	—	—	7/18	—
Juan Jimenez-Cauhe2019	>6 Months	11/41	26/41	4/41	12/41	10/41	2/41
Paulo Müller Ramos2019	6 Months	2/26	16/26	7/26	—	7/26	1/26
R. Pirmez2019	24 Weeks	—	—	—	—	5/25	1/25
Carlos G. Wambier2019	4–6 Months	8/12	4/12	-	8/12	6/12	—
Abhijeet Jha2020	6 Months	14/32	13/32	—	—	—	—
Ratchathorn Panchaprateep2020	24 Weeks	13/30	17/30	—	—	28/30	—
Rita Rodrigues-Barata2020	9 Months	23/148	95/148	30/148	29/148	25/148	1/148
Maria Vastarella2020	24 Weeks	7/12	5/12	—	—	3/12	3/12
Renée A. Beach 2020	3 Months	-	33/51	—	—	22/51	—
Baltazar Sanabria2020	—	—	—	—	—	241/435	26/435
Sergio Vano-Galvan2020	>6 Months	—	20/51	27/51	19/51	14/51	—
Abhijeet Kumar Jha2021	24 Weeks	—	19/47	—	—	36/47	1/47
Sadegh Vahabi-Amlashi2021	9 Months	—	—	—	—	2/26	—
Sergio Van ∼ o-Galvan2021	3 Months	—	—	—	—	212/1404	—
Marcella Nascimento E Silva2022	24 Weeks	2/14	4/14	6/14	—	—	—
C.D. Villarreal-Villarreal2022	3 Months	34/47	9/47	4/47	15/47	—	—
L. Yin2022	9.7 Months	—	—	—	24/60	20/60	5/60
Reese Imhof2023	7.7 Months	—	—	—	6/40	3/40	1/40
Brittany Feaster 2023	≥52 Weeks/12 Months	—	55/105	44/105	10/105	—	—
Matilde Iorizzo2023	—	—	13/15	—	—	—	—
Abena Minta2023	—	5/13	6/13	2/13	4/13	—	—
Ali Asilian2023	6 Months	6/33	13/33	9/33	—	—	—
Leila David Bloch2024	>6 Months	—	—	—	32/100	—	—
Deesha D. Desai2024	—	—	—	—	24/69	—	—
Janaani, P2024	32 Weeks	—	6/22	—	—	—	—
Mariana Alvares Penha2024	24 Weeks	—	—	—	1/45	—	—

### Quality assessment of included studies

We utilized the MINORS scale to assess and score 27 retrospective studies included in our analysis. Among these, fifteen studies were deemed to be of high quality, while twelve were classified as moderate quality. The detailed quality assessment can be found in Supplementary Table S1.

### Efficacy

**FIGURE 2:**
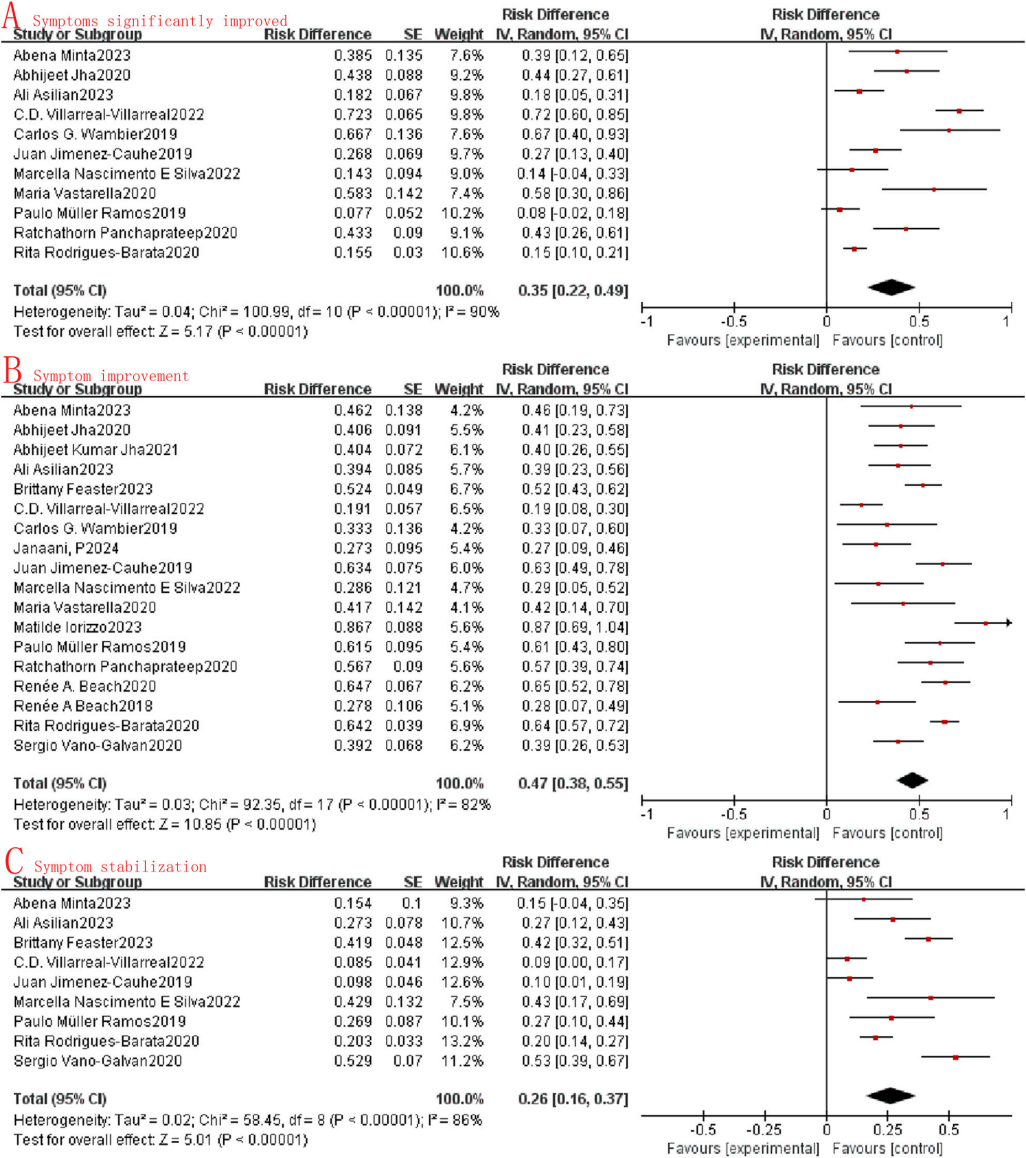
Forest map of the effectiveness of oral minoxidil in the treatment of alopecia. **(A)**: Symptoms significantly improved; **(B)**: Symptom improvement; **(C)**: Symptom stabilization.

This study assessed the efficacy of oral minoxidil in treating alopecia by examining the extent of symptom improvement. Within the collected data, the degree of symptom improvement was categorized as follows: significant symptom improvement, symptom improvement, symptom stability, and symptom deterioration. Owing to the limited data available on symptom deterioration, it was not included in the current statistical analysis. Through the meta-analysis of the included studies, it was observed that the combined odds ratio (OR) for significant symptom improvement, symptom improvement, and symptom stabilization were 0.35 (95%CI: 0.22–0.49, I^2^ = 90%, P < 0.0001, Figure 2A) and 0.47 (95%CI: 0.38–0.55, I^2^ = 82%, P < 0.0001, Figure 2B), and 0.26 (95%CI: 0.16–0.37, I^2^ = 86%, P < 0.0001, Figure 2C). A random effects model was employed due to the substantial heterogeneity (I^2^ > 50%) observed across the studies.

### Safety

**FIGURE 3:**
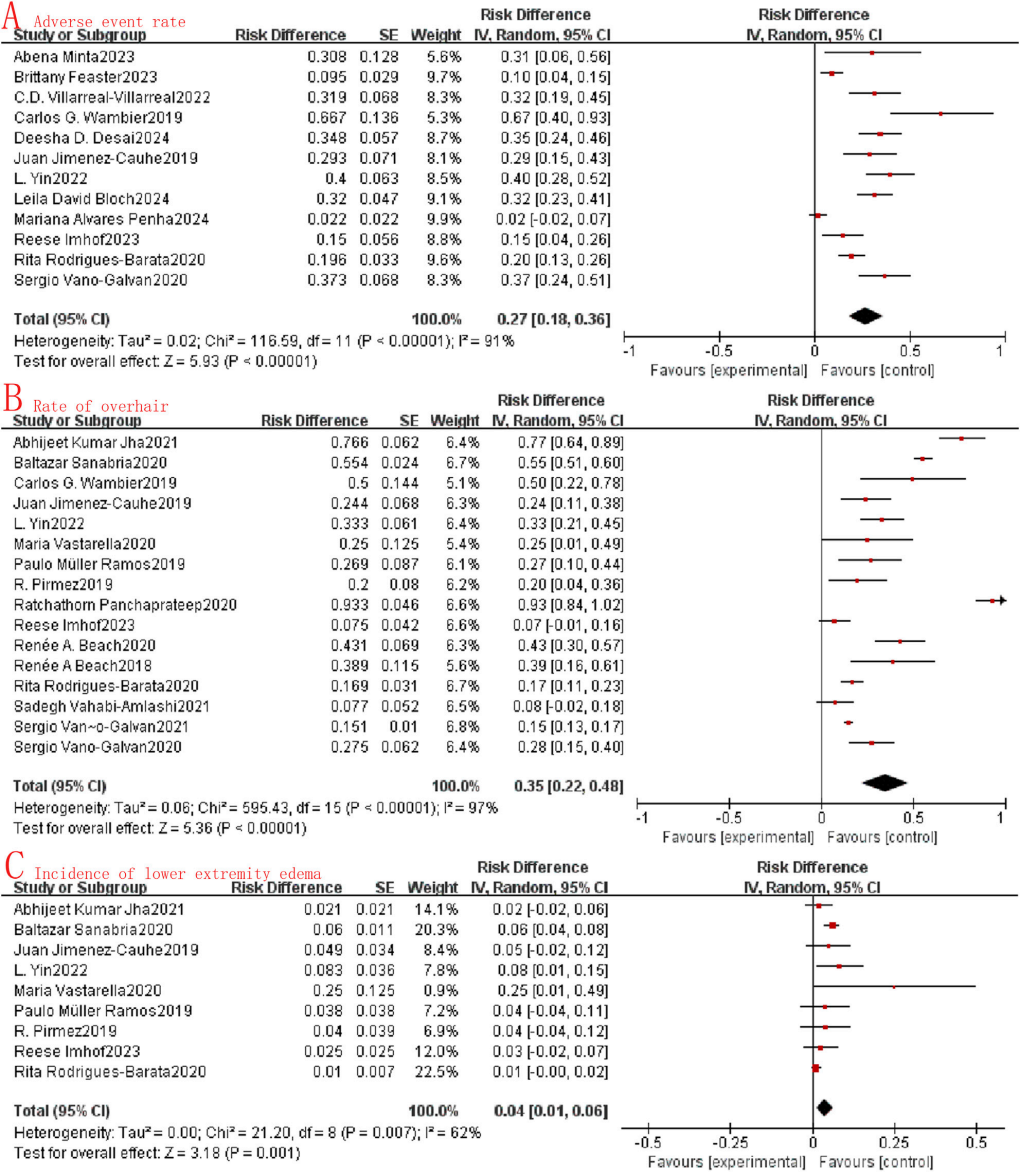
Forest map of the safety of oral minoxidil in the treatment of alopecia. **(A)**: Adverse event rate; **(B)**: Rate of overhair; **(C)**: Incidence of lower extremity edema.

This review revealed that oral minoxidil demonstrated good tolerability, with literature describing only minor adverse effects. Twelve studies reported acceptable safety profiles in relation to adverse events. In our meta-analysis, we observed a combined OR of adverse events at just 0.27 (95%CI: 0.18–0.36, I^2^ = 91%, P < 0.0001, Figure 3A), while the hypertrichosis rate was 0.35 (95%CI: 0.22–0.48, I^2^ = 97%, P < 0.0001, Figure 3B), and the lower limb edema rate was 0.04 (95%CI: 0.01–0.06, I^2^ = 62%, P = 0.001, Figure 3C). These findings collectively support the safety of oral minoxidil in the treatment of alopecia.

### Sensitivity analysis and subgroup analysis

**FIGURE 4:**
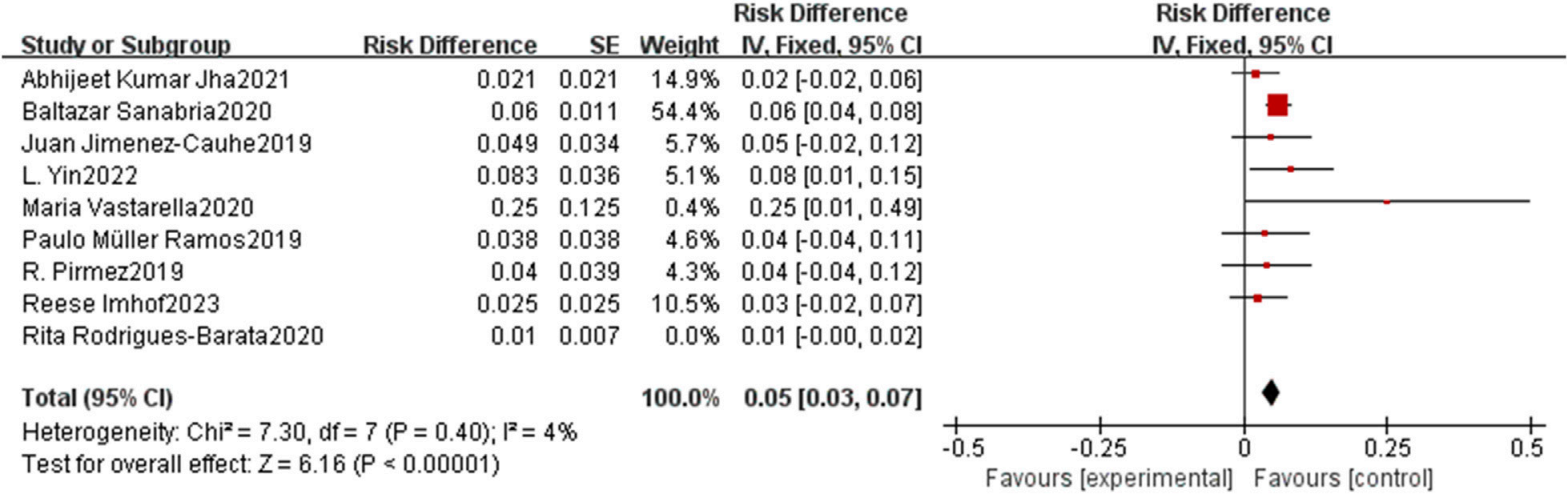
Sensitivity analysis of Incidence of lower extremity edema.

Despite revisiting the study search, selection, and inclusion criteria, heterogeneity remained consistent. To mitigate the potential influence of any particular study, a sensitivity analysis was conducted by reorganizing the included studies. Upon scrutinizing individual studies regarding the incidence of lower limb edema, the investigation highlighted the study by Rodrigues-Barata et al. (2020) as a significant contributor to the observed heterogeneity, despite not being the most heavily weighted among all studies. Remarkably, upon excluding this study, the heterogeneity was notably reduced (P < 0.001, I^2^ = 4%, Figure 4), further affirming the safety of oral minoxidil for alopecia.

**FIGURE 5:**
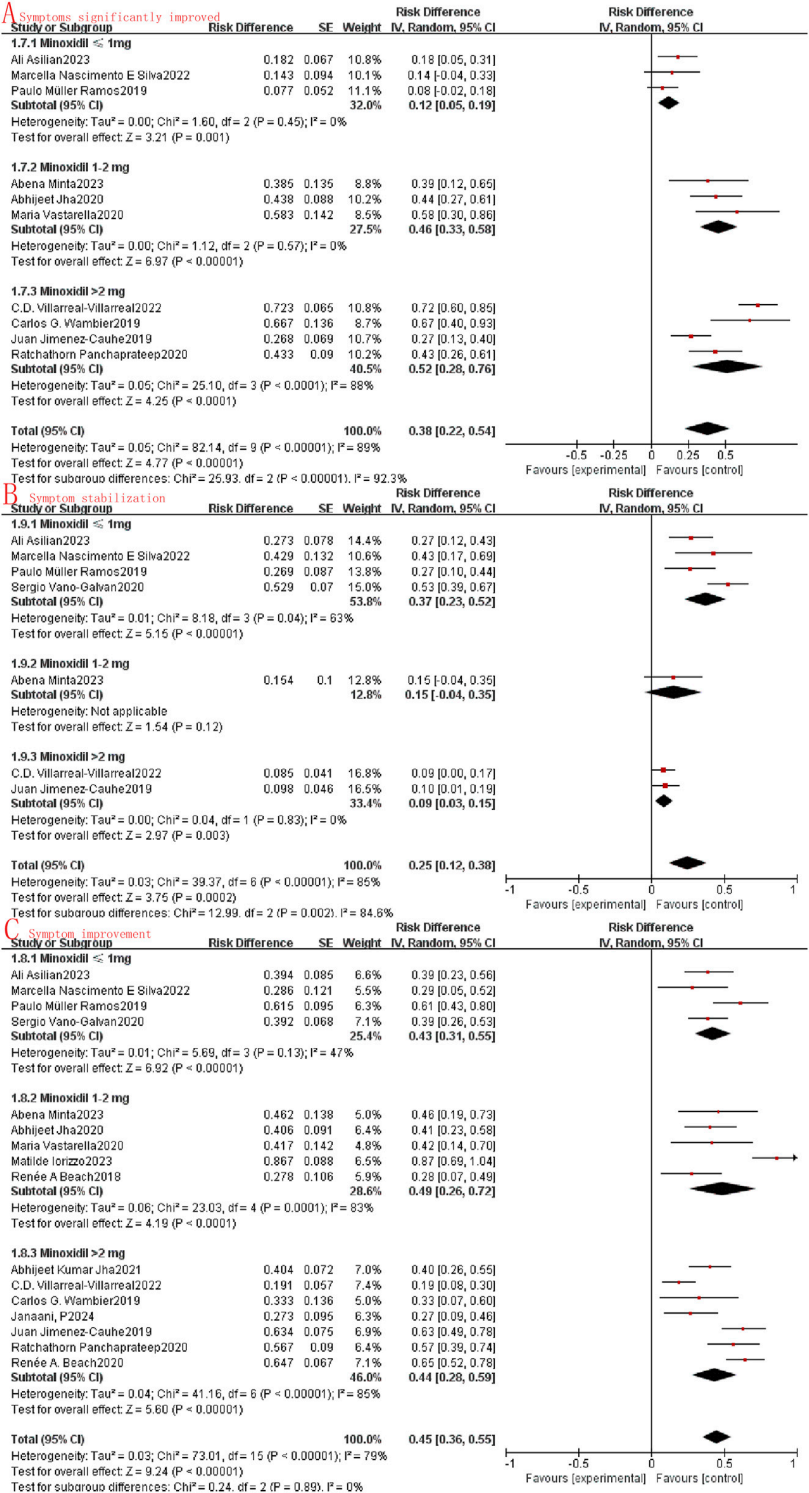
Subgroup analysis **(A)**: Symptoms significantly improved; **(B)**: Symptom stabilization; **(C)**: Symptom improvement.

**FIGURE 6:**
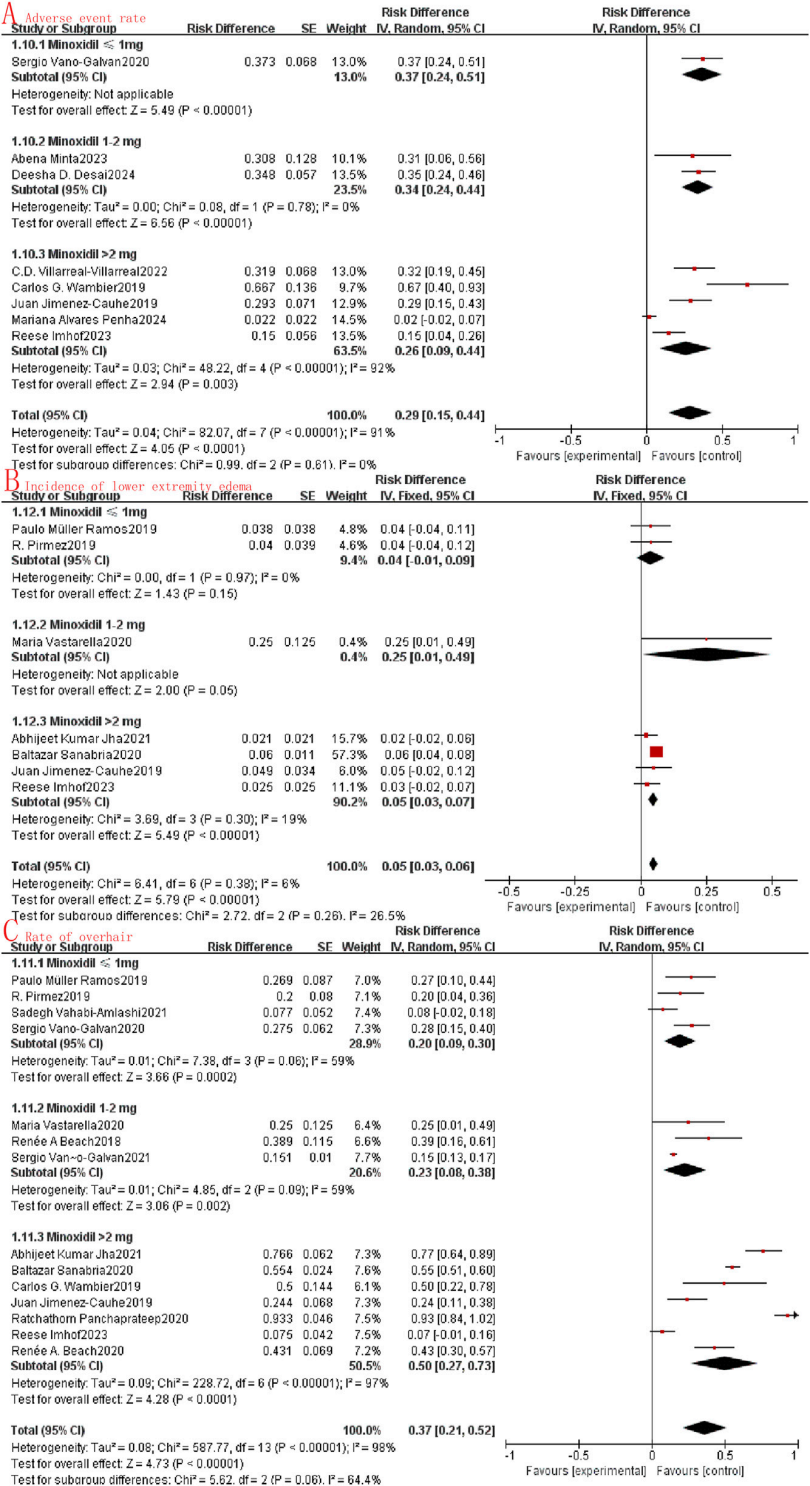
Subgroup analysis **(A)**: Adverse event rate; **(B)**: Incidence of lower extremity edema; **(C)**: Rate of overhair.

Subgroup analysis involves the exploration of potential sources of heterogeneity through random clustering. In our study, we conducted subgroup analyses to assess the impact of oral minoxidil treatment at varying doses. The analysis categorized the study into three groups: the very low dose oral minoxidil group (VLDOM, ≤1 mg), the low dose oral minoxidil group (LDOM, 1–2 mg), and the high dose oral minoxidil group (HDOM, >2 mg). Subgroup analysis showed that the odds ratio between the LDOM and HDOM groups was higher than that of the VLDOM group in terms of symptom improvement (I^2^ = 92.3%, P < 0.001, Figure 5A) and lower than that of the VLDOM group in terms of symptom stabilization (I^2^ = 84.6%, P = 0.002, Figure 5B). However, no significant difference in symptom improvement was noted among the three groups (I^2^ = 0%, P = 0.89, Figure 5C). Additionally, in the subgroup analysis evaluating safety, no statistically significant differences were found in the incidence of adverse reactions (I^2^ = 0%, P = 0.61, Figure 6A) or lower limb edema (I^2^ = 26.5%, P = 0.26, Figure 6B) among the three groups. While the incidence of hypertrichosis in the HDOM group was higher than that in the other two groups (I^2^ = 64.4%, P = 0.06, Figure 6C), the difference was not statistically significant.

## Discussion

In recent years, the utilization of minoxidil to stimulate hair growth has garnered increasing interest. Minoxidil has the ability to directly activate hair follicle growth factors, particularly by stimulating Dermal Papilla and epidermal cells, thus facilitating hair regrowth (Choi et al., 2018; Han et al., 2004). Moreover, minoxidil is believed to expedite the transition of hair follicles from the resting phase to the growth phase by shortening the resting period and prolonging the growth phase. Research indicates that minoxidil can activate the growth of secondary buds in dormant follicles, accelerating their entry into the growth phase (Mori and Uno, 1990). The anti-androgenic property of minoxidil plays a crucial role in combating hair loss, particularly in cases of androgenic alopecia where androgens, notably dihydrotestosterone (DHT), are significant contributors. DHT is known to shrink hair follicles, leading to hair loss. Studies have indicated that minoxidil may impede the expression of 5α-reductase type II, an enzyme responsible for converting testosterone into DHT. By curtailing DHT production, minoxidil can potentially halt or reverse the hair loss process (Gupta et al., 2023; Shen et al., 2023). Furthermore, minoxidil’s anti-androgenic effects may involve modulating the activity of androgen receptors. While the precise mechanism remains incompletely understood, this modulation could help mitigate the adverse impact of androgens on hair follicles (Hsu et al., 2014). Recent research has unveiled that minoxidil may target novel entities such as CYP17A1 and CYP19A1, enzymes linked to androgen synthesis. Through its action on these enzymes, minoxidil can further impede androgen production, bolstering its anti-androgenic attributes (Shen et al., 2023). There is limited safety data on the long-term use of oral minoxidil, but it has been suggested that long-term use may lead to hypotension, tachycardia, and systemic hirsutism. In addition, long-term use may be associated with other health problems such as abnormal kidney function and electrolyte disturbances (Rajab, 2022).

This meta-analysis represents a groundbreaking initiative aimed at assessing the efficacy and safety of oral minoxidil in treating individuals experiencing hair loss. Drawing insights from 27 succinct studies encompassing 2,933 patients, our analysis systematically amalgamated data to quantitatively evaluate the effectiveness and safety of oral minoxidil in addressing alopecia. Among participants, 35% (95% CI: 22%–49%) had significant improvement in symptoms, 47% (95% CI: 38%–55%) had improvement in symptoms, and 26% (95% CI: 16%–37%) had stable symptoms. Subgroup analysis revealed that the odds ratio for symptom improvement was higher in the LDOM group and HDOM group compared to the VLDOM group. The lack of a clear difference in the significance of symptom improvement between the low and high dose groups may be due to the fact that the dose-response curve of minoxidil reaches a “minimum effective dose” within a certain range, and further increases in the dose do not significantly enhance the efficacy of the treatment. Moreover, the response to minoxidil may vary among patients depending on baseline follicular status, vascular function, metabolic profile, and other factors. In the low-dose group, some patients may have approached or achieved optimal efficacy, whereas in the high-dose group, some patients may not have achieved better results due to individual differences (e.g., slower metabolism rates) (Olsen et al., 2002). Additionally, the odds ratio for symptom stabilization was lower in the HDOM group than in the VLDOM group. However, there was no significant variance in symptom improvement observed among the three groups. In our meta-analysis, we found that the combined odds ratio (OR) for adverse events was notably low at 0.27. Specifically, the incidence rates for hypertrichosis and lower limb edema were recorded at 0.35 and 0.04, respectively. Moreover, within the safety evaluation subgroup analysis, no statistical significance was observed in the incidence of adverse reactions, lower limb edema, and hirsutism across the three groups. Oral Minoxidil acts on hair follicles through systemic blood circulation, not just locally. This systemic absorption may lead to the activation of hair follicles in other parts of the body as well, thus triggering hirsutism. Hair follicles in different areas may have different sensitivities to minoxidil. High doses of minoxidil may activate hair follicles that are normally dormant, resulting in excessive hair growth. Minoxidil may also indirectly promote hair growth by affecting hormone levels (e.g., androgens), especially in female patients (Williams et al., 2024; Gupta et al., 2022; Vañó-Galván et al., 2021b). Certainly, among the studies we analyzed, two studies within each of the three groups reported instances of hypotension. The occurrence of hypotension was minimal in each group, and no participants withdrew from the study due to this issue.

Oral minoxidil has exhibited promising efficacy in addressing hair loss; however, to enhance treatment outcomes, the significance of combination therapy should not be overlooked. Research indicates that combining minoxidil with finasteride may enhance treatment efficacy. Finasteride, a 5α-reductase inhibitor primarily used for male pattern alopecia, has been found to complement minoxidil effectively. A study revealed that the use of 0.1% topical finasteride alongside 5% minoxidil contributed to maintaining hair density, especially in individuals previously treated with oral finasteride. This combined approach offers a new treatment avenue for patients who do not respond adequately to monotherapy. Spironolactone, an anti-androgen medication frequently prescribed for female pattern hair loss, has been found effective in combating this condition. A study indicated that the combination of 0.25 mg oral minoxidil with 25 mg spironolactone led to a noteworthy reduction in hair loss and enhancement in hair density (Devjani et al., 2023; Sadeghzadeh Bazargan et al., 2024). When considering the combination of these two medications, the patient’s overall health and past medical history need to be considered first. Drug interactions are also key considerations. Spironolactone may interact with a variety of medications, including, but not limited to, certain antibiotics, antihypertensives, and antidepressants. In addition, patients should have their liver and kidney function and electrolyte levels monitored regularly while using these medications. By integrating the mechanisms and impacts of various medications, physicians can provide patients with a more holistic and tailored approach to addressing hair loss. Subsequent research endeavors ought to delve deeper into identifying the most advantageous combinations to enhance the efficacy of hair loss interventions and elevate the quality of life for individuals grappling with this concern.

### Limitations

The meta-analysis is subject to several limitations. Firstly, a significant portion of the included articles are in the form of Letters, potentially impacting the reliability and methodological rigor of the data. Secondly, the absence of key indicators and the lack of randomized clinical trials represent significant shortcomings. Additionally, the evaluation of effectiveness is influenced by individual subjectivity, underscoring the need for more precise values for statistical analysis. Furthermore, the effect of oral minoxidil may be influenced by the type of hair loss, warranting further analysis in additional studies. Lastly, there is inadequate systematic reporting of long-term prognostic effects.

## Conclusion

This systematic review and meta-analysis indicate that individuals with hair loss may derive benefits from oral minoxidil, particularly at doses exceeding 1 mg. Nonetheless, additional research or data is essential to definitively establish its efficacy and safety.

## Data Availability

The original contributions presented in the study are included in the article/Supplementary Material, further inquiries can be directed to the corresponding author.

## References

[B1] Asilian A. Farmani A. Saber M. (2024). Clinical efficacy and safety of low-dose oral minoxidil versus topical solution in the improvement of androgenetic alopecia: a randomized controlled trial. J. Cosmet. dermatology 23 (3), 949–957. 10.1111/jocd.16086 38031516

[B2] Beach R. A. (2018). Case series of oral minoxidil for androgenetic and traction alopecia: tolerability and the five C's of oral therapy. Dermatol. Ther. 31 (6), e12707. 10.1111/dth.12707 30246901 PMC6586015

[B3] Beach R. A. McDonald K. A. Barrett B. M. Abdel-Qadir H. (2020). Tolerated, effective, successful: low dose oral minoxidil for treating alopecia, A 3-year north American retrospective case series. J. Am. Acad. Dermatology 84, e239–e240. 10.1016/j.jaad.2020.12.038 34756848

[B4] Bloch L. D. Carlos R. M. D. (2024). Side effects' frequency assessment of low dose oral minoxidil in male androgenetic alopecia patients. Skin. appendage Disord., 1–5. 10.1159/000539969 PMC11793886 39911974

[B5] Burzi L. Alessandrini A. M. Quaglino P. Piraccini B. M. Dika E. Ribero S. (2021). Cutaneous events associated with immunotherapy of melanoma: a review. J. Clin. Med. 10 (14), 3047. 10.3390/jcm10143047 34300213 PMC8308045

[B6] Choi N. Shin S. Song S. U. Sung J. H. (2018). Minoxidil promotes hair growth through stimulation of growth factor release from adipose-derived stem cells. Int. J. Mol. Sci. 19 (3), 691. 10.3390/ijms19030691 29495622 PMC5877552

[B7] Devine B. L. Fife R. Trust P. M. (1977). Minoxidil for severe hypertension after failure of other hypotensive drugs. Br. Med. J. 2 (6088), 667–669. 10.1136/bmj.2.6088.667 902045 PMC1631896

[B8] Devjani S. Ezemma O. Kelley K. J. Stratton E. Senna M. (2023). Androgenetic alopecia: therapy update. Drugs 83 (8), 701–715. 10.1007/s40265-023-01880-x 37166619 PMC10173235

[B9] Dika E. Patrizi A. Ribero S. Fanti P. A. Starace M. Melotti B. (2016). Hair and nail adverse events during treatment with targeted therapies for metastatic melanoma. Eur. J. dermatology EJD 26 (3), 232–239. 10.1684/ejd.2016.2747 27019511

[B10] Feaster B. Onamusi T. Cooley J. E. McMichael A. J. (2023). Oral minoxidil use in androgenetic alopecia and telogen effluvium. Archives dermatological Res. 315 (2), 201–205. 10.1007/s00403-022-02331-5 35244759

[B11] Gan D. C. Sinclair R. D. (2005). Prevalence of male and female pattern hair loss in Maryborough. J. investigative dermatology Symposium Proc. 10 (3), 184–189. 10.1111/j.1087-0024.2005.10102.x 16382660

[B12] Gilmore E. Weil J. Chidsey C. (1970). Treatment of essential hypertension with a new vasodilator in combination with beta-adrenergic blockade. N. Engl. J. Med. 282 (10), 521–527. 10.1056/NEJM197003052821001 4391708

[B13] Gupta A. Hall D. Talukder M. Bamimore M. (2022). There is a positive dose-dependent association between low-dose oral minoxidil and its efficacy for androgenetic alopecia: findings from a systematic review with meta-regression analyses. Skin. appendage Disord. 8, 355–361. 10.1159/000525137 36161084 PMC9485924

[B14] Gupta A. K. Talukder M. Shemer A. Piraccini B. M. Tosti A. (2023). Low-dose oral minoxidil for alopecia: a comprehensive review. Skin. appendage Disord. 9 (6), 423–437. 10.1159/000531890 38376087 PMC10806356

[B15] Hagstam K. E. Lundgren R. Wieslander J. (1982). Clinical experience of long-term treatment with minoxidil in severe arterial hypertension. Scand. J. urology Nephrol. 16 (1), 57–63. 10.3109/00365598209179641 7089494

[B16] Hair loss (2005). Epidemiological approach. J. Am. Acad. Dermatology 52 (3), P104.

[B17] Han J. H. Kwon O. S. Chung J. H. Cho K. H. Eun H. C. Kim K. H. (2004). Effect of minoxidil on proliferation and apoptosis in dermal papilla cells of human hair follicle. J. dermatological Sci. 34 (2), 91–98. 10.1016/j.jdermsci.2004.01.002 15033191

[B18] Hsu C. L. Liu J. S. Lin A. C. Yang C. H. Chung W. H. Wu W. G. (2014). Minoxidil may suppress androgen receptor-related functions. Oncotarget 5 (8), 2187–2197. 10.18632/oncotarget.1886 24742982 PMC4039155

[B19] Imhof R. Villalpando B. Torgerson R. (2023). 42482 Low dose oral minoxidil as monotherapy in patients with hair loss. J. Am. Acad. Dermatology 89 (3), AB191. 10.1016/j.jaad.2023.07.765

[B20] Iorizzo M. Waśkiel-Burnat A. Anedda J. Piraccini B. M. Apalla Z. Rudnicka L. (2023). Persistent chemotherapy-induced alopecia treated with low dose oral minoxidil: a multicenter retrospective case series of 15 patients. Dermatology Pract. and Concept. 13 (3), e2023152. 10.5826/dpc.1303a152 PMC10412018 37557121

[B21] Janaani P. Kumaran S. Vinay K. Mehta H. Sharma V. K. (2024). Comparative efficacy of low-dose oral minoxidil, topical minoxidil, and platelet-rich plasma with topical minoxidil in androgenetic alopecia: a randomized controlled trial. Br. J. Dermatology 192, i68. 10.1007/s00403-025-03938-0 39954138

[B22] Jha A. K. Sonthalia S. Zeeshan V. K. (2020). Efficacy and safety of very-low-dose oral minoxidil 1.25 mg in male androgenetic alopecia. J. Am. Acad. Dermatology 83 (5), 1491–1493. 10.1016/j.jaad.2020.05.129 32492469

[B23] Jha A. K. Zeeshan M. Singh A. Roy P. K. (2021). Platelet-rich plasma with low dose oral minoxidil (1.25mg versus 2.5mg) along with trichoscopic pre- and post-treatment evaluation. J. Cosmet. dermatology 20 (10), 3220–3226. 10.1111/jocd.14049 33682223

[B24] Jimenez-Cauhe J. Saceda-Corralo D. Rodrigues-Barata R. Hermosa-Gelbard A. Moreno-Arrones O. M. Fernandez-Nieto D. (2019). Effectiveness and safety of low-dose oral minoxidil in male androgenetic alopecia. J. Am. Acad. Dermatology 81 (2), 648–649. 10.1016/j.jaad.2019.04.054 31054970

[B25] Minta A. Park C. Rose L. Trovato S. Dulmage B. (2023). Retrospective review of oral and topical minoxidil for cancer treatment-induced hair loss. Archives dermatological Res. 315 (9), 2613–2615. 10.1007/s00403-023-02660-z 37421421

[B26] Mori O. Uno H. (1990). The effect of topical minoxidil on hair follicular cycles of rats. J. dermatology 17 (5), 276–281. 10.1111/j.1346-8138.1990.tb01641.x 2380431

[B27] Nascimento e Silva M. Ramos P. M. Silva M. R. Nascimento e Silva R. (2022). Barbosa Raposo NR: **randomized clinical trial of low-dose oral minoxidil for the treatment of female pattern hair loss: 0.25 mg versus 1 mg** . J. Am. Acad. Dermatology 87 (2), 396–399. 10.1016/j.jaad.2022.01.017 35077777

[B28] Olsen E. A. Dunlap F. E. Funicella T. Koperski J. A. Swinehart J. M. Tschen E. H. (2002). A randomized clinical trial of 5% topical minoxidil versus 2% topical minoxidil and placebo in the treatment of androgenetic alopecia in men. J. Am. Acad. Dermatology 47 (3), 377–385. 10.1067/mjd.2002.124088 12196747

[B29] Page M. J. McKenzie J. E. Bossuyt P. M. Boutron I. Hoffmann T. C. Mulrow C. D. (2021). The PRISMA 2020 statement: an updated guideline for reporting systematic reviews. J. Clin. Epidemiol. 134, 178–189. 10.1016/j.jclinepi.2021.03.001 33789819

[B30] Panchaprateep R. Lueangarun S. (2020). Efficacy and safety of oral minoxidil 5 mg once daily in the treatment of male patients with androgenetic alopecia: an open-label and global photographic assessment. Dermatology Ther. 10 (6), 1345–1357. 10.1007/s13555-020-00448-x PMC7649170 32970299

[B31] Penha M. A. Miot H. A. Kasprzak M. Müller Ramos P. (2024). Oral minoxidil vs topical minoxidil for male androgenetic alopecia: a randomized clinical trial. JAMA dermatol. 160 (6), 600–605. 10.1001/jamadermatol.2024.0284 38598226 PMC11007651

[B32] Pirmez R. Salas-Callo C.-I. (2020). Very-low-dose oral minoxidil in male androgenetic alopecia: a study with quantitative trichoscopic documentation. J. Am. Acad. Dermatology 82 (1), E21–E22. 10.1016/j.jaad.2019.08.084 31520662

[B33] Rajab F. (2022). Low-dose oral minoxidil for hair growth. Dermatol. Times 43 (11), 38–39.

[B34] Ramos P. M. Sinclair R. D. Kasprzak M. Miot H. A. (2019). Minoxidil 1 mg oral versus minoxidil 5% topical solution for the treatment of female-pattern hair loss: a randomized clinical trial. J. Am. Acad. Dermatology 82, 252–253. 10.1016/j.jaad.2019.08.060 31473295

[B35] Rodrigues-Barata R. Moreno-Arrones O. M. Saceda-Corralo D. Jimenez-Cauhe J. Ortega-Quijano D. Fernandez-Nieto D. (2020). Low-dose oral minoxidil for female pattern hair loss: a unicenter descriptive study of 148 women. Skin. appendage Disord. 6 (3), 175–176. 10.1159/000505820 32656239 PMC7325226

[B36] Sadeghzadeh Bazargan A. Tavana Z. Dehghani A. Jafarzadeh A. Tabavar A. Alavi Rad E. (2024). The efficacy of the combination of topical minoxidil and oral spironolactone compared with the combination of topical minoxidil and oral finasteride in women with androgenic alopecia, female and male hair loss patterns: a blinded randomized clinical trial. J. Cosmet. dermatology 23 (2), 543–551. 10.1111/jocd.15979 37650533

[B37] Sanabria B. Vanzela T. N. Miot H. A. Ramos P. M. (2021). Adverse effects of low-dose oral minoxidil for androgenetic alopecia in 435 patients. J. Am. Acad. Dermatology 84 (4), 1175–1178. 10.1016/j.jaad.2020.11.035 33253848

[B38] Shen Y. Zhu Y. Zhang L. Sun J. Xie B. Zhang H. (2023). New target for minoxidil in the treatment of androgenetic alopecia. Drug Des. Dev. Ther. 17, 2537–2547. 10.2147/DDDT.S427612 PMC10461613 37645625

[B39] Vahabi-Amlashi S. Layegh P. Kiafar B. Hoseininezhad M. Abbaspour M. Khaniki S. H. (2021). A randomized clinical trial on therapeutic effects of 0.25 mg oral minoxidil tablets on treatment of female pattern hair loss. Dermatol. Ther. 34 (6), e15131. 10.1111/dth.15131 34529341

[B40] Vañó-Galván S. Pirmez R. Hermosa-Gelbard A. Moreno-Arrones Ó. M. Saceda-Corralo D. Rodrigues-Barata R. (2021b). Safety of low-dose oral minoxidil for hair loss: a multicenter study of 1404 patients. J. Am. Acad. Dermatology 84 (6), 1644–1651. 10.1016/j.jaad.2021.02.054 33639244

[B41] Vañó-Galván S. Trindade de Carvalho L. Saceda-Corralo D. Rodrigues-Barata R. Kerkemeyer K. L. Sinclair R. D. (2021a). Oral minoxidil improves background hair thickness in lichen planopilaris. J. Am. Acad. Dermatology 84 (6), 1684–1686. 10.1016/j.jaad.2020.04.026 32289397

[B42] Vastarella M. Cantelli M. Patri A. Annunziata M. C. Nappa P. Fabbrocini G. (2020). Efficacy and safety of oral minoxidil in female androgenetic alopecia. Dermatol. Ther. 33 (6), e14234. 10.1111/dth.14234 32851744

[B43] Villarreal-Villarreal C. D. Boland-Rodriguez E. Rodriguez-Leon S. Le Voti F. Vano-Galvan S. Sinclair R. D. (2022). Dutasteride intralesional microinjections in combination with oral minoxidil vs. oral minoxidil monotherapy in men with androgenetic alopecia: a retrospective analysis of 105 patients. J. Eur. Acad. Dermatology Venereol. 36 (7), E570–E572. 10.1111/jdv.18066 35279887

[B44] Wambier C. G. Craiglow B. G. King B. A. (2021). Combination tofacitinib and oral minoxidil treatment for severe alopecia areata. J. Am. Acad. Dermatology 85 (3), 743–745. 10.1016/j.jaad.2019.08.080 31499158

[B45] Wang H. Pan L. Wu Y. (2022). Epidemiological trends in alopecia areata at the global, regional, and national levels. Front. Immunol. 13, 874677. 10.3389/fimmu.2022.874677 35911734 PMC9331164

[B46] Williams K. N. Olukoga C. T. Y. Tosti A. (2024). Evaluation of the safety and effectiveness of oral minoxidil in children: a systematic review. Dermatology Ther. 14 (7), 1709–1727. 10.1007/s13555-024-01197-x PMC11264662 38861138

[B47] Yin L. Svigos K. Gutierrez D. Peterson E. Lo Sicco K. Shapiro J. (2022). Low-dose oral minoxidil increases hair density and thickness in androgenetic alopecia: a retrospective analysis of 60 patients. J. Eur. Acad. Dermatology Venereol. JEADV 36 (3), e200–e202. 10.1111/jdv.17731 34637178

