# Supplementary material for: Efficacy and safety of oral minoxidil in the treatment of alopecia: a single-arm rate meta-analysis and systematic review
Source: Front Pharmacol. 2025 Jun 3;16:1556705. doi: 10.3389/fphar.2025.1556705 (PMC12188453; doi:10.3389/fphar.2025.1556705)
Supplement: Supplementary file 1 [file Table1.docx]

**Supplementary Table 1** Assessment of non-randomized controlled trials in the version of MINORS.

| Study | A clearly  stated  aim | Inclusion of  consecutive  patients | Prospective  collection of  data | Endpoint  appropriate  to the study  aim | Unbiased  assessment  of endpoints | Follow-up  period  appropriate  to the major  endpoint | Loss to  follow  up not  exceeding  5% | Prospective  calculation  of the study  size | Total score |
| --- | --- | --- | --- | --- | --- | --- | --- | --- | --- |
| Renée A Beach2018 | 2 | 2 | 1 | 1 | 2 | 1 | 2 | 0 | 11 |
| Juan Jimenez-Cauhe2019 | 2 | 2 | 2 | 2 | 2 | 1 | 2 | 2 | 15 |
| Paulo Müller Ramos2019 | 2 | 2 | 2 | 1 | 2 | 1 | 2 | 1 | 13 |
| R. Pirmez2019 | 2 | 2 | 1 | 1 | 2 | 1 | 2 | 1 | 12 |
| Carlos G. Wambier2019 | 2 | 2 | 2 | 1 | 2 | 1 | 2 | 0 | 12 |
| Abhijeet Jha2020 | 2 | 2 | 1 | 1 | 2 | 1 | 2 | 1 | 12 |
| Ratchathorn Panchaprateep2020 | 2 | 2 | 1 | 1 | 2 | 1 | 2 | 1 | 12 |
| Rita Rodrigues-Barata2020 | 2 | 2 | 2 | 2 | 2 | 1 | 2 | 2 | 15 |
| Maria Vastarella2020 | 2 | 2 | 2 | 1 | 2 | 1 | 2 | 0 | 12 |
| Renée A. Beach 2020 | 2 | 2 | 1 | 1 | 2 | 1 | 2 | 2 | 13 |
| Baltazar Sanabria2020 | 2 | 2 | 1 | 1 | 2 | 1 | 2 | 2 | 13 |
| Sergio Vano-Galvan2020 | 2 | 2 | 2 | 1 | 2 | 1 | 2 | 2 | 14 |
| Abhijeet Kumar Jha2021 | 2 | 2 | 1 | 1 | 2 | 1 | 2 | 2 | 13 |
| Sadegh Vahabi-Amlashi2021 | 2 | 2 | 1 | 1 | 2 | 1 | 2 | 1 | 12 |
| Sergio Van~o-Galvan2021 | 2 | 2 | 1 | 1 | 2 | 1 | 2 | 2 | 13 |
| Marcella Nascimento E Silva2022 | 2 | 2 | 1 | 1 | 2 | 1 | 2 | 0 | 11 |
| C.D. Villarreal-Villarreal2022 | 2 | 2 | 2 | 1 | 2 | 1 | 2 | 2 | 14 |
| L. Yin2022 | 2 | 2 | 1 | 1 | 2 | 1 | 2 | 2 | 13 |
| Reese Imhof2023 | 2 | 2 | 1 | 1 | 2 | 1 | 2 | 2 | 13 |
| Brittany Feaster 2023 | 2 | 2 | 1 | 1 | 2 | 1 | 2 | 2 | 13 |
| Matilde Iorizzo2023 | 2 | 2 | 1 | 1 | 2 | 1 | 2 | 0 | 11 |
| Abena Minta2023 | 2 | 2 | 2 | 1 | 2 | 1 | 2 | 0 | 12 |
| Ali Asilian2023 | 2 | 2 | 1 | 1 | 2 | 1 | 2 | 1 | 12 |
| Leila David Bloch2024 | 2 | 2 | 1 | 1 | 2 | 1 | 2 | 2 | 13 |
| Deesha D. Desai2024 | 2 | 2 | 1 | 1 | 2 | 1 | 2 | 2 | 13 |
| Janaani, P2024 | 2 | 2 | 1 | 1 | 2 | 1 | 2 | 1 | 12 |
| Mariana Alvares Penha2024 | 2 | 2 | 1 | 1 | 2 | 1 | 2 | 2 | 13 |
